# Stochastic Simulations of Normal Aging and Werner’s Syndrome

**DOI:** 10.1007/s11538-014-9952-8

**Published:** 2014-04-26

**Authors:** Qi Qi, Jonathan A. D. Wattis, Helen M. Byrne

**Affiliations:** 1 Centre for Mathematical Medicine and Biology, School of Mathematical Sciences, University of Nottingham, Nottingham , NG7 2RD UK; 2 Mathematical Institute, University of Oxford, Andrew Wiles Building, Radcliffe Observatory Quarter, Woodstock Road, Oxford, OX2 6GG UK

**Keywords:** Telomere dynamics, End-replication problem, Aging, Werner’s syndrome, Stochastic simulation

## Abstract

Human cells typically consist of 23 pairs of chromosomes. Telomeres are repetitive sequences of DNA located at the ends of chromosomes. During cell replication, a number of basepairs are lost from the end of the chromosome and this shortening restricts the number of divisions that a cell can complete before it becomes senescent, or non-replicative. In this paper, we use Monte Carlo simulations to form a stochastic model of telomere shortening to investigate how telomere shortening affects normal aging. Using this model, we study various hypotheses for the way in which shortening occurs by comparing their impact on aging at the chromosome and cell levels. We consider different types of length-dependent loss and replication probabilities to describe these processes. After analyzing a simple model for a population of independent chromosomes, we simulate a population of cells in which each cell has 46 chromosomes and the shortest telomere governs the replicative potential of the cell. We generalize these simulations to Werner’s syndrome, a condition in which large sections of DNA are removed during cell division and, amongst other conditions, results in rapid aging. Since the mechanisms governing the loss of additional basepairs are not known, we use our model to simulate a variety of possible forms for the rate at which additional telomeres are lost per replication and several expressions for how the probability of cell division depends on telomere length. As well as the evolution of the mean telomere length, we consider the standard deviation and the shape of the distribution. We compare our results with a variety of data from the literature, covering both experimental data and previous models. We find good agreement for the evolution of telomere length when plotted against population doubling.

## Introduction

While aging is a natural and inevitable feature of all living organisms, the mechanisms that regulate it and determine an individual’s lifespan remain to be fully elucidated. Improvements in diet, environment, medical care, and the development of science and technology, even over the last century, have contributed to an increase in the average human lifespan from 45 to 75 years (Kirkwood 2005). When trying to understand these changes, in addition to considering external factors, we should also consider those processes which occur inside the cells and organs of the human body and limit lifespan.

In order to understand aging, it is natural to consider what happens to an individual cell and its progeny and how the changes associated with aging are affected by processes occurring at the subcellular level (Kirkwood and Austad 2000). Biologically, there are many processes which occur on the cellular level which contribute to aging at the organism level. For example, there is the accumulation of oxidative damage, the appearance of nucleotide mutations during DNA replication, and, during cell division, a number of basepairs are lost from one end of a chromosome due to incomplete replication of the DNA strand: this is known as the end replication problem. Much research into aging has its origins in the pioneering work by Hayflick and Moorehead (1961) who, in 1961, discovered that cells have a limited capacity for proliferation.

The DNA in the nucleus of each human cell is partitioned into 46 chromosomes, a chromosome being a large coiled structure comprising a single piece of DNA. The regions of repetitive DNA at each end of the chromosome are called telomeres (Cooper and Hausman 2009): they protect chromosomes from losing genetic material and prevent chromosome fragments rejoining. We view aging as a telomere shortening process since it appears that telomere length determines whether a cell can divide and is a key factor in determining a cell’s potential for proliferation. When the telomere length is critically short, the chromosome stops replicating (Olovnikov 1971), and the cell becomes senescent. In this paper, we focus on the role of telomere shortening and the onset of senescence as a contributory factor in aging. We are not claiming that this is the sole factor involved in the aging process of organisms, merely that in the scenarios we model, we assume that other aging processes can be ignored, and that the aging effects observed are due to telomere shortening.

At present, there is no consensus about how telomere shortening occurs. It is possible that the amount of telomere lost when a single chromosome replicates and the probability that a chromosome divides may vary. For example, chromosomes with longer telomeres may lose more basepairs and/or have a greater probability of dividing than those with shorter telomeres (Buijs et al. 2004; Portugal et al. 2008).

In this paper, we consider various mechanisms for how much telomere is lost per replication, and alternative formulae for the probability of replication in a given time interval (which we term “generation”). We develop a mathematical model to compare the effect that each rule has on the dynamics of telomere shortening in a population of cells. We consider the model in various subcases, in Case A, a chromosome (cell) divides whenever its telomeres are long enough to allow division. In Case B, there is a probability of each chromosome (cell) dividing. Each of these cases is then further subdivided into two subcases: in Case A1, every generation all chromosomes with sufficiently long telomeres divide, and one of each offspring loses a fixed amount telomere. In Case A2, this rule is generalised so that the amount of telomere lost depends on telomere length; and again, every chromosome divides during each generation provided its telomere is sufficiently long. In Case B1, a fixed amount of telomere is lost in each replication event, but there is a probability of division depending on telomere length. Case B2 generalises cases A2 and B1 in that both the rate of telomere loss and the probability of cell division are dependent on telomere length. In each case, we are interested in investigating how the average telomere length of the chromosomes and the proportion of dividing chromosomes (or cells) changes with generation number. Clearly model B2 will provide the best fit to any data, since it incorporates all other models as special cases. However, attempting to fit the other models to data will help determine which factors influence the evolution of the distribution of telomere lengths. There is little experimental evidence on the precise factors which determine the rate of loss of telomere. Thus one reason for proposing a variety of models in this paper is to allow the hypotheses that (i) telomere length influences cell division and (ii) the rate of telomere loss depends on telomere length can be tested against data. Note these hypotheses are not mutually exclusive.

Similar rules for telomere length changes over generations have been considered by other authors, including Cases A1, A2, and B1. In Levy et al. (1992) studied Case A1, and predicted that average telomere length decreases linearly with generation number; they also found the fraction of dividing chromosomes. Our results for Case A1 are consistent with those presented in Levy et al. (1992). In Buijs et al. (2004) analyzed Case A2, with telomere loss linearly dependent on telomere length. They fitted experimental data of Martens et al. (2000) and Zhang et al. (2000) on the distribution of telomere lengths, verifying that a model in which telomere shortening depends on telomere length is consistent with the experimental data. In Portugal et al. (2008) considered Case B1 where telomere loss is fixed, but the probability of division depends on telomere length; however, in our Case B1, as well as predicting the average telomere length and the fraction of senescent cells, we also consider the probability of cells replicating being a nonlinear function of telomere length. Our work on Case B2 where both telomere loss and cell division probability depend on telomere length, is new, as is our study of Werner’s syndrome (see below).

An obvious weakness of the above models is that chromosomes are treated as a single population, in which individual chromosomes undergo division independently. In reality, they are in groups of $N=46$ to cell (in humans), and all 46 must replicate synchronously when a cell divides. Hence, in this paper, we proceed to generalise the models to the cell-level where we consider a population of cells, each composed of $N=46$ chromosomes, and a cell replicates only if *all* of its chromosomes are able to replicate. This represents a significant generalisation of the existing models. Putting $N=1$ would return us to the model of independent chromosomes considered earlier, which can thus be thought of as a special case of the cell-level model.

Werner’s syndrome is an inherited disease characterized by rapid aging. In their second or third decade patients normally develop gray hair, wrinkled skin, alopecia, diabetes mellitus, and juvenile cataracts (Yamamoto et al. 2003). The average lifespan for Werner’s syndrome patients is about 45 years and their deaths are often linked to malignant tumors (Goto et al. 1996). The limited lifespan of Werner’s syndrome patients is caused by large, spontaneous deletions of DNA, which lead to accelerated telomere loss and attenuated apoptosis (Faragher et al. 1993). Proliferation of cells is often measured in population doublings (pd), this relates the number of cells $N(t)$ at some time $t$, to an initial value $N(0)=N_0$ by $pd=\log _2(N(t)/N_0)$. Fibroblasts from Werner’s syndrome patients only undergo approximately 20 population doublings, which is 40 population doublings less than normal human fibroblasts. While the molecular mechanisms underpinning Werner’s syndrome are unknown, several hypotheses have been proposed, including the mutator phenotype. Here, the Werner’s syndrome patient develops chromosomal aberrations, deletions (Wyllie et al. 2000; Furuichi 2001), and a higher somatic mutation rate (Fukuchi et al. 1989). There is strong evidence that Werner’s syndrome accelerates a cell’s journey to senescence. Experiments reported in Tahara et al. (1997) have shown dramatic shortening of telomeres in Werner’s syndrome fibroblasts and B-lymphoblastoid cells, and that senescence happens faster than in normal fibroblasts and B-lymphoblastoid cells. This suggests that dramatic telomere shortening can accelerate cell senescence.

Tahara et al. (1997) reports that when a population of Werner’s syndrome cells becomes senescent, the range of telomere lengths, namely 3,500 to 18,500 basepairs (bp) is much wider than that from a population of normal cells (5,500–9,000 bp). A possible explanation for this is that the cells of Werner’s syndrome patients contain some chromosomes which have critically short telomeres and others with much longer ones. Telomere dysfunction is caused by critically short telomeres, which, in the absence of recombination, trigger premature cell senescence (Chang 2005). Another hypothesis is that a mutation in the Werner’s syndrome gene plays an important role in Werner’s syndrome (Bachrati and Hickson 2003), due to its role in DNA maintenance and repair.

In this paper, we model Werner’s syndrome by assuming that the cell suffers an extra loss of telomere when it divides (Opresko et al. 2003). Thus, we treat Werner’s syndrome as an accelerated version of the normal aging model by considering a variety of amounts of telomere loss per replication and a range of probabilities of this additional loss occurring. The remainder of this paper is organized as follows. In Sect. 2, we propose rules for replication of chromosomes in the case of normal aging and develop an algorithm using Monte-Carlo simulations. After presenting the results of this work, in Sect. 3 we generalise the algorithm by scaling the model up to a population of dividing cells undergoing division in which each cell contains $N=46$ chromosomes (the number of chromosomes in a normal human cell). Finally, in Sect. 4, we form the replication rule for Werner’s syndrome, Monte Carlo simulations are undertaken and we compare the dynamics predicted from our models with that for Werner’s syndrome. Conclusions are drawn and discussed in Sects. 5 and 6.

## Normal Aging: Model Development

### Single Chromosome

**Fig. 1:**
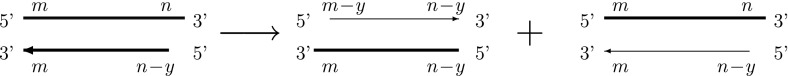
Diagram showing how telomere length in an individual chromosomes changes during replication. *Thick lines* correspond to the template (or parent) strands and *thin lines* represent replicated strands of the template associated with each daughter chromosome. The *arrows* indicate the direction in which replication takes place

In this section, we develop a mathematical model of normal aging based on the biological processes summarized in Fig. 1. We consider individual chromosomes which divide independently of each other. In order for our explanation to be consistent with later work (Sect. 3), we introduce the model, including the terms “cells” and “chromosomes,” and although we initially consider cells to contain just one chromosome, we will later relax this assumption so that each cell has 46 chromosomes. The two strands of the DNA double helix are not reversible, that is, they have a direction. The two ends of a DNA strand are distinct, one being referred to as the 3’ end and the other as the 5’ end. This terminology refers to the carbon atom in the deoxyribose molecule involved in attachment of the next phosphate group. Replication occurs in the 5’ $\rightarrow$ 3’ direction. During replication the double stranded DNA separates, with one strand going to each daughter cell. Each strand is used as a template for the construction of a complementary strand. However, the creation of this secondary strand is incomplete, leading to a shortening of one end, and a consequent reduction in telomere length.

The mathematical models presented in this paper are based on a number of assumptions. First, there is no telomere elongation during replication, that is, we neglect telomerase activity and assume that there are no recombination events. Secondly, cells can only exist in one of in two states: a dividing state or a senescent state. When a cell becomes senescent, it remains in that state: it cannot start dividing again. We do not account for cell death. Thirdly, we use the term “generation number” to mean iteration number, that is the timescale over which cells have the opportunity to divide once; in our algorithm (mathematical model) this is the iteration number and corresponds to the timescale of interest. In many experiments, instead of this fixed timescale, population doubling is used to measure the evolution of the process (Harley and Goldstein 1980). A population doubling corresponds to the total number of cells doubling. The time for a population doubling to occur varies as cells age: typically population doublings occur more quickly at the start of experiments and slow down later. Generation number and population doubling are similar at the start of simulations or experiments, when all cells are able to divide. However, at later stages of the experiment or simulation, the time for one population doubling is longer than the time for one generation as a greater proportion of cells are senescent and so fewer cells contribute to the growth of the population.

**Fig. 2:**
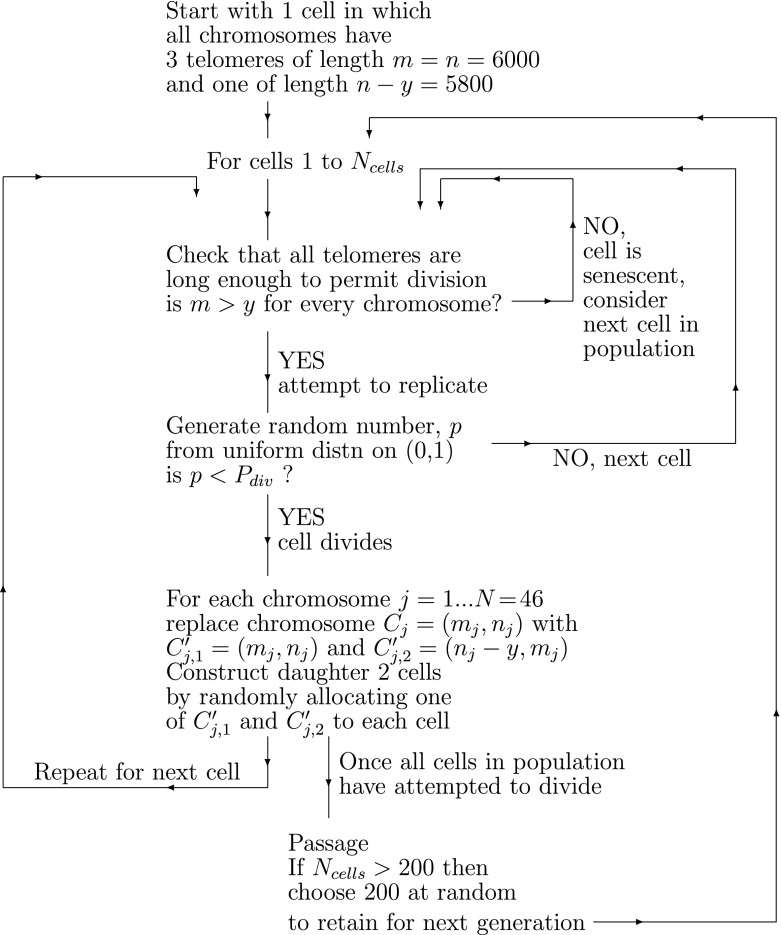
Flowchart illustrating algorithm used to simulate cell division and track the associated evolution of telomere lengths

Initially, we suppose that telomere shortening is caused only by the end replication problem. We assume that normal chromosome replication produces one chromosome which is identical to its parent and one which is slightly shorter (see Fig. 1). The algorithm is summarized in Fig. 2, where $P_{div}$ is the probability of a nonsenescent cell undergoing division. We use an initial telomere length of 6,000 bps. This is a typical value for human telomeres, and is used by Buijs et al. (2004) and Iwama et al. (1998). Although experimental measurements of telomere length are significantly longer, they include a threshold length below which telomeres cannot shorten. We subtract this threshold from our measurement of length, so that our threshold length is zero.

In more detail, suppose that a chromosome has two telomeres lengths with $m$ and $n$ base pairs on one strand, denoted $(m,n)$, the other strand having telomeres of length $m$ and $n-y$, or $(m,n-y)$. One of these parent strands will be passed to each daughter cell. Each strand is used as a template for DNA replication, however, this process is imperfect, and results in incomplete replication. We assume that $y$ base pairs are lost per replication so that the $(m,n)$ parent strand gains a complementary strand with telomeres of length $(m,n-y)$, thus producing a daughter chromosome identical to its parent. The shorter parent, having telomeres of lengths $(m,n-y)$ yields a complementary strand with lengths $(m-y,n-y)$. This process is illustrated in Fig. 1 and, following Arino et al. (1995), can be written more compactly as follows:

1 $$\begin{aligned} \underbrace{\left( \begin{array}{cc} m &{} n\\ m &{} n-y\end{array} \right) }_{\hbox {parent}} \rightarrow \underbrace{\left( \begin{array}{cc} m-y &{} n-y\\ m &{} n-y\end{array}\right) }_{\mathop {\hbox {(shorter)}}\limits _{\hbox {daughter}}} + \underbrace{ \left( \begin{array}{cc} m &{} n\\ m &{} n-y\end{array}\right) }_{\mathop {\hbox {(identical)}}\limits _{\hbox {daughter}}}. \end{aligned}$$

In practice, the number of basepairs lost ($y$) and the probability that a chromosome divides ($P_{div}$) may vary with telomere length. For example, chromosomes with longer telomeres may lose more basepairs than those with shorter ones and chromosomes with longer telomeres may have a greater probability of dividing than those with shorter telomeres. We account for these effects by assuming that $y(n)$ is linearly dependent on $n$ and $P_{div}=P_{div}(n)$ where

2 $$\begin{aligned} y(n)=y_{0}+y_1n\,,\quad P_{div}(n)=\left( a+bn\right) ^\alpha \,, \end{aligned}$$

see Buijs et al. (2004) and Portugal et al. (2008) for a discussion of these alternatives. In (2), $y_0$ represents the amount of telomere lost each generation, and $y_1$ is a constant of proportionality, giving a greater loss for long telomeres than short ones (since we expect $y_1>0$); $y_{0}$, $y_1$, $a$ and $b$ are constants and $\alpha$ is a parameter with $0 \le \alpha \le 1$. The case $\alpha =1$ was considered by Portugal et al. in 2008. In order to see clearly how changes in average telomere length depend on telomere loss, $y(n)$, and the probability that a chromosome divides, $P_{div}(n)$, we consider the four cases outlined in Table 1. For Case B2, both the rate of telomere loss and the probability of replication depend on telomere length. If the parameters $y_{0},y_1, \alpha , a, b$ are chosen appropriately, Cases A1, A2, and B1 can be considered as special cases of Case B2: Case A1 is recovered by setting $y_1=0$ and $\alpha =0$; Case A2 is obtained by fixing $\alpha =0$; and Case B1 by setting $y_1=0$. Simulation results for the four cases are presented below.

### Case A ($P_{div}=1$)

Here, on each time step, any cell that can divide will do so, as $P_{div}=1$. The amount of telomere lost during replication is $y=y_0+ny_1$. For case A1, we assume that the amount of telomere lost is independent of telomere length, so that $y = y_0 = 200$ basepairs (bps) and $y_1=0$. For a fair comparison with Case A1, in Case A2 we choose a length-dependent loss rate of the form $y(n)=100+n /30$ per generation, so that when $n=3,000$, the loss rate is 200, as in Case A1. In Case A2, the loss is composed of two terms: a fixed loss term (100 bps) and a term which is directly proportional to telomere length ($n/30$ bps), so that longer telomeres are shortened at a higher rate and shorter telomeres at a lower rate.

**Table 1:** Summary of the four models of telomere shortening under consideration

Case	Prob of division $P_{div}(n) = (a+bn)^\alpha$	Telomere loss $y(n)=y_0+ny_1$	Explanation (reference)
A1	$$P_{div}(n)=1$$	$$y(n)=200 $$	Loss and division are constant (Levy et al. 1992)
A2	$P_{div}(n)=1$	$y(n) = 100 \!+\! n/30$	Length-dependent loss (Buijs et al. 2004)
B1	$P_{div}(n) = (n\!-\!200) / 5750$	$y(n) = 414$	Length-dependent division probability (Portugal et al. 2008)
B2	$P_{div}(n)=(n\!-\!200) / 5750$	$y(n) = 207 \!+\! n/14$	Length-dependent loss and division

At each generation, we record not only the average telomere length but also the number of cells that have just replicated. We denote by $N(g)$ the number of cells at generation $g$. The quantity $\phi _{div}(g)$ represents the fraction of dividing cells at generation $g$ and $\phi _{sen}(g)$ the fraction of senescent cells at generation $g$, so that

3 $$\begin{aligned} \phi _{div}(g-1)=\frac{N(g)-N(g-1)}{N(g-1)}, \quad \phi _{sen}(g)=1-\phi _{div}(g). \end{aligned}$$

### Results for Case A

**Fig. 3:**
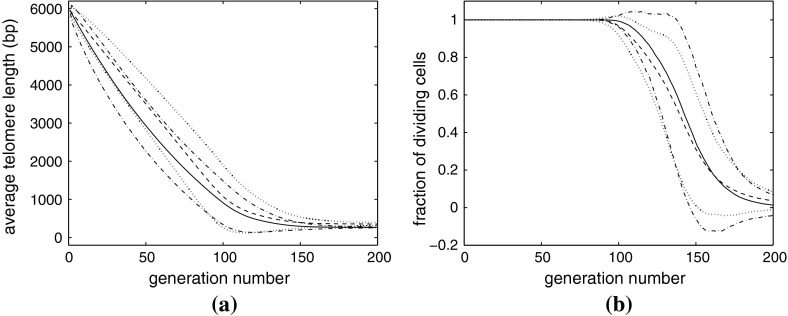
Average results of 1,000 realizations of our model of normal aging, when cases A1 and A2 are considered; **a** plot of average telomere length against generation number; **b** the fraction of dividing cells plotted against generation number. The average telomere length initially shortens more slowly for Case A1 ($y(n)=200$, mean shown by *solid line*, *dotted lines* indicate mean $\pm$2 sd) than for Case A2 ($y(n)=100+n/30$, mean indicated by *dashed line*, *dash-dotted lines* show mean $\pm$2 sd). At later times, when the average telomere length has fallen below 3,000 bps, the telomeres shorten more slowly for Case A2 than A1. After about 170 generations, both populations are completely senescent and their telomere lengths do not change

In Fig. 3a, we show how the average telomere length changes with generation number for Cases A1 and A2. As expected, the average telomere length initially decreases *more* rapidly for Case A2 (solid line) than for Case A1 (dashed line), this persists until the length falls below 3000 bps; the average telomere length then decreases more *slowly* for Case A2 than Case A1. After generation 170, the average telomere length for both models is similar as both populations are senescent.

The results presented in Fig. 3b reveal a similar trend in the proportion of replicative cells. In particular, until about generation 80, all cells in both models divide because no cells have yet become senescent. Thereafter, Case A1 has a lower proportion of replicating cells than Case A2. By generation 170 the fractions are similar because almost all cells are senescent. Between generations 80 and 170 the standard deviation (sd) of Case A2 is greater than that of Case A1 because more basepairs are deleted per division for Case A2.

At first sight it might seem counterintuitive that the cells with a *shorter* mean telomere length (A2 in panel a) have a *higher* rate of cell division (panel b). This situation arises because it is not the *mean* telomere length that governs division, but the number of telomeres above threshold, and the distribution of telomere lengths is more widely spread in case A2 than A1. Note that some of the mean $\pm$2 sd curves lie outside the physically relevant region of $\phi _{div}\in (0,1)$. This is due to the distribution of telomere lengths being non-normal at the start and of the transition to senescence. For example, at the end of the process, most chromosomes have the same minimum telomere length, while there are a few with significantly longer telomeres, but none with telomeres shorter than the minimum. Thus, the distribution will be skewed.

### Case B: Chromosome Division Dependent on Telomere Length

For case A, we distinguish two types of cells: those that can divide and those which are senescent. In contrast, for Case B, we have *three* types of cell: (i) cells that divided in the generation $(g-1)\rightarrow g$; (ii) cells that were already senescent at generation $(g-1)$; and (iii) cells that *could* have divided at generation $(g-1)\rightarrow g$ but did not do so. We remark that these cells are not senescent since their telomeres are sufficiently long that they could divide at a later time.

We denote by $N(g)$ the number of cells at generation $g$ and by $\phi _{div }(g)$ the fraction of cells that divided at generation $g$, so that $\phi _{div }(g)$ is as given in (3). At each generation, we monitor the telomere length of all cells and use this to determine $N_s(g)$, the number of cells senescent at generation $g$, that is, those cells that cannot divide because their chromosome is too short. Then $N(g)-N_s(g)$ represents the number of cells which could divide at the next generation. We denote by $\phi _{sen}(g)$ the fraction of senescent cells at generation $g$ and by $\phi _{pot}(g)$ the proportion of cells which had the potential to divide, but did not do so at generation $g$, so that,

4 $$\begin{aligned} \phi _{sen}(g)=\frac{N_s(g)}{N(g)}\,,\quad \phi _{pot}(g)=1-\phi _{div}(g)-\phi _{sen}(g), \end{aligned}$$

and this replaces the definition of $\phi _{sen}$ in (3).

The simulations presented in this section are similar to those presented in Sect. 2.2. We start with a single chromosome with average telomere length of 5,950 basepairs (that is, three telomeres of length 6,000 and one of 5,800), and passage its progeny to senescence. In order to compare results with Case A1, we first choose the simpler case, B1, in which the number of basepairs lost per replication is length-*in*dependent, while the probability of division is length-dependent. We assume $P_{div}=(n-200)/5750$, so that initially, when $n=5,950$, $P_{div}=1$ and when one of the telomeres reaches the threshold length for senescence (200 bps), $P_{div}=0$. We suppose that $y_0=414$ bps are lost per replication, so that when the chromosomes are half-way to senescence (at $n=2,975$), the expected number of basepairs lost per division ($y_0P_{div}$) is 200 basepairs, as for Case A1.

### Results for Case B1

**Fig. 4:**
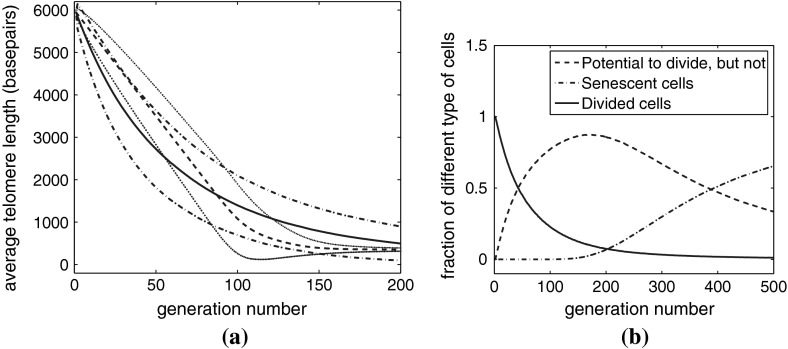
Average results of 1000 simulations for Case B1 with $y(n)=414$ and $P_{div}(n)=(n-200)/5750$. **a** the *solid* and *dashed lines* are the average telomere length plotted against generation for Cases B1 and A1, respectively, with two standard deviations above and below the mean indicated by *dash-dotted* and *dotted lines*. **b** The *dash-dotted line* indicates the fraction of senescent cells $\phi _{sen}$ plotted against generation number. The *solid line* indicates the fraction of cells which divided $\phi _{div }$ in the previous generation. The *dashed line* indicates the fraction of cells $\phi _{pot}$ which have the potential to divide, but did not divide in the previous generation

In Fig. 4a, the average telomere length in Case B1 is shown initially to decrease faster with generation number than in Case A1. This is because for Case B1, the loss rate ($y(n)=414$ bps per division) is more than twice the value used for Case A1, and near the start of the simulation most chromosomes are sufficiently long that they have a high likelihood of dividing.

Although the probability of replication, $P_{div}$, decreases with telomere length, before generation 50, $P_{div}>1/2$, so the *average* telomere loss for Case B1 ($P_{div}y(n)$) is greater than that for Case A1 (which is only 200 basepairs). At later times, as the telomeres shorten in length, the average loss rate in Case B1 falls below that for Case A1. At approximately generation 90, the two curves cross, and thereafter the average telomere length for Case B1 exceeds that for Case A1. At later generations, Case B1 exhibits markedly slower convergence to senescence than Case A1. The standard deviation of telomere length initially increases in a similar fashion in both cases, but at later times, in Case B1 only decreases a little, whereas the standard deviation of Case A1 reduces significantly. This is due to the slower onset of senescence for Case B1.

In Fig. 4b we show how for Case B1 the proportion of each cell type changes with generation number. The fraction of nonsenescent cells consists of those which divided $(\phi _{div})$ and those which did not divide but could have done so, $(\phi _{pot})$. We note that $\phi _{div}$ decreases and that $\phi _{sen}$ increases over time. Further, $\phi _{pot}$, initially increases with generation number, attains a maximum between generations 150 and 200, and then declines to zero.

### Results for Case B2

For Case B2, since the probability of a chromosome dividing varies with telomere length, we distinguish three types of chromosomes, as for Case B1, using Eqs. (3)–(4) to calculate $\phi _{div}$, $\phi _{sen}$, and $\phi _{pot}$. As for Case B1, we fix $P_{div}(n) = (n-200)/5750$, so that $P_{div}=1$ when the average telomere length is $n=5,950$ and $P_{div}=0$ when the average telomere length reaches the threshold value of $n=200$. We use a loss rate of $y(n)=207+n/14$, so that half-way to senescence, when $n=2975$, $P_{div}=0.487$ and $y(n)=420$ so that the expected telomere loss is $P_{div}(n)y(n)=205$ basepairs, approximately the same value as for Case A1. A summary of the parameter values considered is presented in Table 1.

**Fig. 5:**
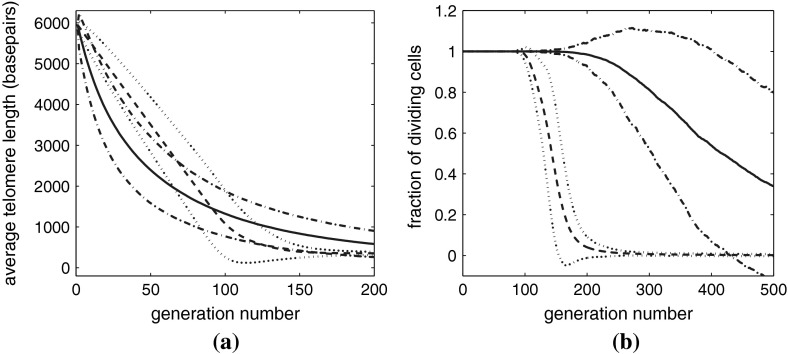
Comparison of dynamics predicted for Cases A1 ($P_{div}=1$, $y(n)=200$) and B2 ($P_{div}=(n-200)/5750$, $y(n)=207+n/14$) obtained by averaging results from 1,000 simulations of each case. **a** For Case B2, (mean $=$ *solid line*, $\pm$2 sd $=$ *dash-dotted line*), the average telomere length decreases more rapidly than for Case A1, the standard deviations do not rapidly decay to zero. **b** Case B2 shows a gradual transition to senescence as the mean fraction of nonsenescent cells, $1-\phi _{sen}(n)$, decreases significantly more slowly than for Case A1, which shows a sharp transition around generation 150

In Fig. 5a, we compare the dynamics of telomere shortening associated with cases A1 and B2. Initially, the average telomere length for Case B2 (solid line) decreases significantly faster than in Case A1 (dashed line). However, at later times, Case B2 approaches senescence more slowly than Case A1. This is because the probability of division for nonsenescent cells and the rate of basepairs on division are both small. For example, in Case B2, when the telomere length reaches 1,500 bps, (3/4 of the way to senescence), $P_{div}(n)= 0.278$ and $Y(n)=314$ bps, so the average telomere loss is only 87 bps, less than half the loss rate associated with Case A1.

The proportion of each type of chromosome ($\phi _{div}$, $\phi _{pot}$, $\phi _{sen}$) changes with generation number in a similar fashion to Case B1 (results not shown). The difference between Cases A1 and B2 is more noticeable in Fig. 5b, where we compare $1-\phi _{sen}$, the fraction of nonsenescent cells. For Case A1, between generations 1–80 all cells divide; after that, some cells become senescent and the fraction of nonsenescent cells decreases rapidly from unity to zero. For Case B2, the fraction of nonsenescent cells $\phi _{div} + \phi _{pot}$ remains unity until generation 170, about twice as long as in Case A1; and then decreases only slowly. Since both the probability of division and the amount of telomere lost decrease, as the generation number increases, the approach to senescence is extremely slow.

## Cell Model

### Preliminaries

In Sect. 2, we considered a population of individual chromosomes. In practice, however, division occurs at the *cellular* level and cells typically contain many chromosomes, this number varying between species. For example, small deer have 6 chromosomes, while carp contain over 100 (Alberts et al. 2008). Since there are 46 chromosomes in normal human cells, in this section, we fix $N=46$ to denote the number of chromosomes in a cell. We start with a single cell, fixing $n=6,000$ basepairs for each of its $N=46$ chromosomes.

*Check for senescence* Before a cell replicates, we check that none of its telomeres have fallen below the critical value. If one of the chromosomes has reached the critical value, then the cell will not replicate and is classed as senescent.

*Check for cell division* Each chromosome obeys the replication rule (1). For stochastic simulations, we keep track of the length of each chromosome in each cell. In order to present meaningful results, we report the results of simulations using $C_m^g$ to denote a cell with *total* telomere length $m$ at generation $g$. Thus, if $n_j(g)$ (with $j=1,\ldots ,N$) are the lengths of individual chromosomes in a cell, the total telomere length is $m=\sum _{j=1}^{N}n_j$.

We assume further that if a cell can divide, its probability of undergoing division depends on the mean telomere length via $P_{div}(\overline{n}) = (a+b\overline{n})^\alpha$ where $a\,b$ and $\alpha$ are constants and $\overline{n}=m/N$. As in Sect. 2 we consider the four cases of telomere shortening introduced in Table 1.

*Rules for division* As before we continue to apply length-dependent telomere loss for each replication, with $y(n)=y_{0}+y_{1}n$ where $y_{0}$ and $y_{1}$ are constants. If a cell replicates, it produces two daughter cells, with the parent chromosome providing one chromosome for each daughter cell, although these chromosomes are allocated to the daughter cells independently and randomly. In consequence, there are $2^{N}$ ways in which the $2N$ daughter chromosomes can be allocated to the two daughter cells.

*Check  for  passaging.* We use the passaging method outlined in Sect. 2.1, that is, if the number of cells exceeds 200, we randomly select 200 from the full population to track in the next generation; this makes our simulation method similar to the experimental procedure of passaging. We assume that the rest of the population has similar telomere length properties as the retained subpopulation. Thus at each generation we record the telomere lengths in each of 200 cells and plot not only the average telomere length of chromosomes in the cells but also the shortest telomere length of the 46 chromosomes in each cell.

In the following subsection, we present results for Case A1 in detail, and in Sect. 3.3 we summarize and compare the results for all four cases.

### Results for Case A1 ($y(n)=200$, $P_{div}=1$)

**Fig. 6:**
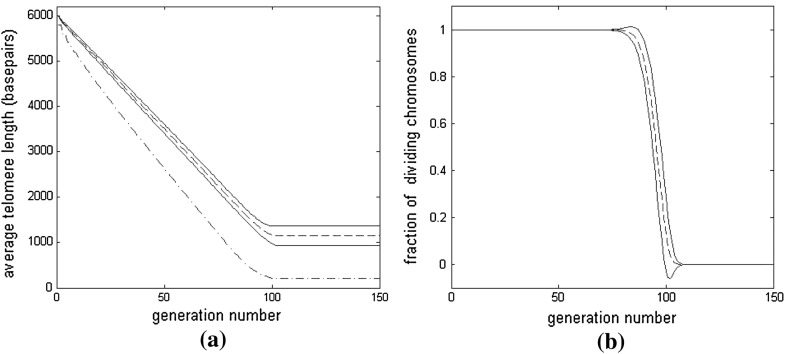
Results for Case A1, where the loss term is $y(n)=y_0=200$ bps and cells divide whenever possible, that is, $P_{div}=1$. Averages are taken over 1,000 simulations. **a** The *dashed line* shows average telomere length of the chromosome plotted against generation number, the *solid lines* above and below are the average telomere length plus and minus twice the standard deviation, respectively. The *dash-dot line* shows the average length of the shortest telomere in each cell. **b** The fraction of dividing cells plotted against generation number, plus/minus two standard deviations

In Fig. 6, we present averaged results from 1000 simulations in order to show how the average telomere length changes with generation number. Figure 6a reveals that as the generation number increases from 1 to 90, the mean telomere length of the cells decreases linearly, while the standard deviation increases but remains small. After generation 90, all curves undergo a sharp transition and plateau at constant values; the mean telomere length being about 1,100 bps. Figure 6a also shows that the average length of the *shortest* telomere in each cell decreases linearly but at a slightly faster rate than that at which the average telomere length decreases. The shortest telomere reaches the critical length of 200 basepairs at about generation 100 causing the population to become senescent with an average telomere length of 1,100 basepairs; as the shortest telomere reaches the critical value, the whole cell stops replicating. In Fig. 6b we show how the fraction of dividing cells changes with generation number: between generations 90 and 100, all cells become senescent.

**Fig. 7:**
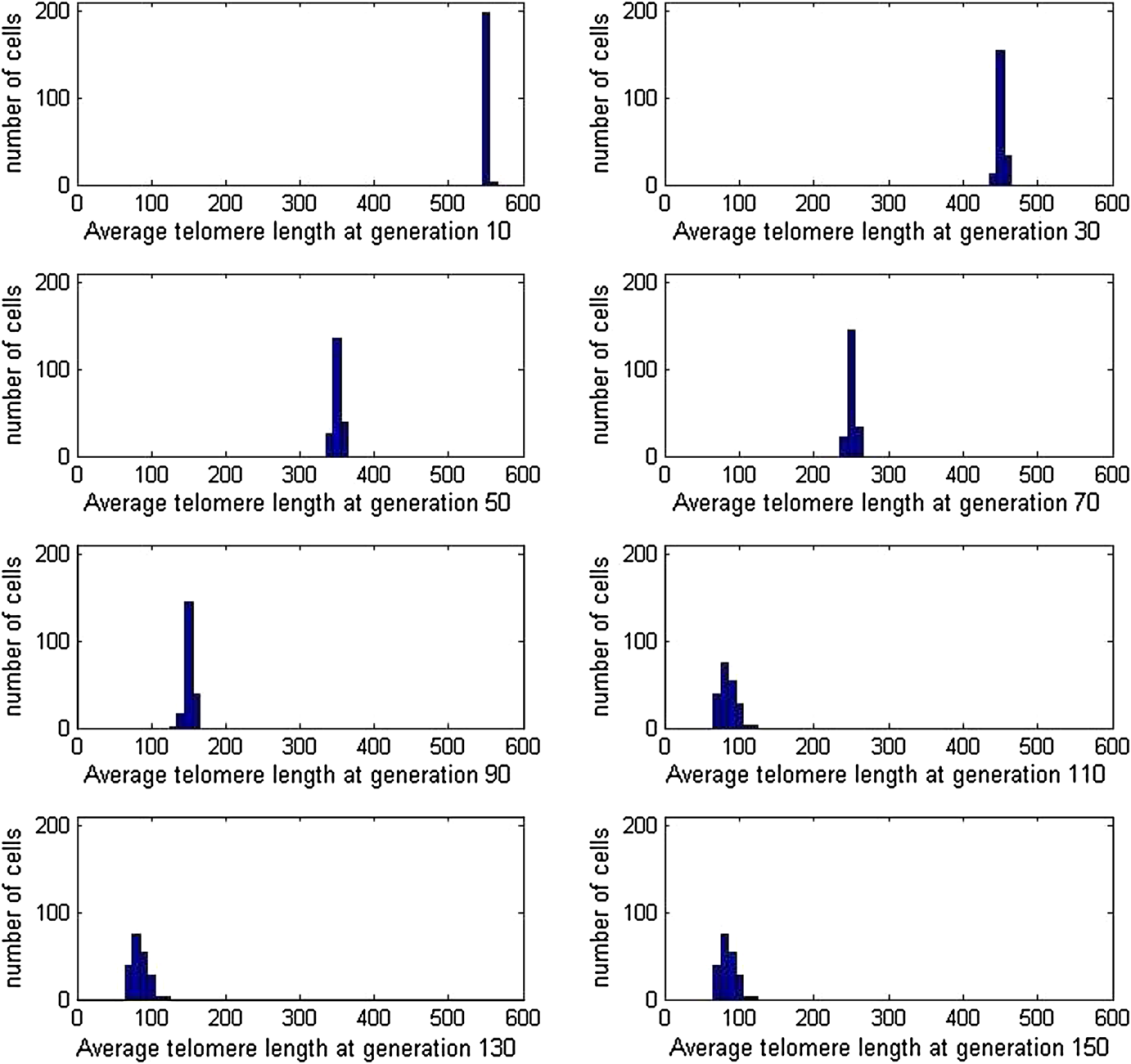
At generations 10, 30, 50, 70, 90, 110, 130, 150, we plot *histograms* showing the distribution of average telomere lengths from a sample of 200 cells chosen at random. The cells replicate according to Case A1. Note that for clarity, the horizontal scale (average telomere length) has been reduced by a factor of 10

In order to understand better how senescence arises, in Fig. 7 we show how the distribution of telomere lengths from a particular simulation or realization changes with generation number. As the generation number increases the distribution spreads out as it moves to the left, towards shorter telomere lengths. The results for generations 110, 130, 150 are identical, indicating that the cells here became senescent.

### Comparison of Results for Cases A and B

In this section, we present simulation results similar to those presented in Sect. 3.2. We start with a single cell containing 46 chromosomes, each with 3 telomeres of length 6000, and one of length 5,800 bps. We follow its progeny to senescence. In our previous simulations (see Sects. 2.3, 2.5, 2.6), we assumed that the chromosome division probability $P_{div}$ was either constant ($\alpha =0$ in equation (2); Case A) or linearly dependent on telomere length ($\alpha =1$ in Eq. (2); Case B). In this section, we consider cases for which $0 \le \alpha \le 1$. For Cases A1 and B1, the amount of telomere lost per replication is fixed, that is $y_1=0$ and the parameters $a$, $b$, and $y_{0}$ in (2) are chosen to ensure that the average telomere loss per chromosome replication is 200 basepairs. We fix $\alpha =0$, 0.25, 0.5, 0.75, 1 separately with a constant telomere loss of $y(n)=y_0$, whereas for Cases A2 and B2 telomere loss is length-dependent, $y(n)$. The parameter values are listed in Table 2, and give approximate telomere losses of 200 basepairs per chromosome replication when the system is half-way between the initial conditions and senescence.

**Table 2:** The parameter values for the 10 different cases, each of which has an expected telomere loss of about 200 bps per chromosome replication when half-way to senescence ($\overline{n}=3,000$)

Case	$\alpha$	$y_0$	$y_1$
Case A1	0	200	0
Case A2	0	100	1/30
Case B1.1	0.25	240	0
Case B1.2	0.5	288	0
Case B1.3	0.75	345	0
Case B1.4	1	414	0
Case B2.1	0.25	120	1/25
Case B2.2	0.5	144	1/21
Case B2.3	0.75	172.5	1/17
Case B2.4	1	207	1/14

The loss is given by $y(n) = y_0+n y_1$ and the probability of division is $P_{div} = (a+b\overline{n})^\alpha = ((\overline{n}-200)/5750)^\alpha$, that is, $a=-200/5750$ and $b=1/5750$

As before, for Case A1, the cell always divides if all telomeres of all chromosomes exceed the critical length (that is, $P_{div}=1$), and the loss of telomere is constant (200 basepairs). Case A2 also has $P_{div}=1$, but telomere loss in each replication depends on telomere length, *via* $y=y_0+n y_1$ with $y_0,y_1$ specified in Table 1.

For Case B1, the number of basepairs lost per replication is fixed ($y_0>0$, $y_1=0$), whereas the probability of replication is telomere length dependent with $0 < P_{div}(n) < 1$. We assume $P_{div}(\overline{n}) = (a+b\overline{n})^\alpha$ with $a=1/5750$ and $b=-200/5750$ as in Table 1. Here $\overline{n}$ is the mean telomere length over the cell. We consider four different values of $\alpha$ and $y_0$, (see Table 2); the values of $\alpha$ being chosen to ensure that when the mean telomere length is $\overline{n} = 2975$ bps, the expected loss $P_{div}(\overline{n}) y(\overline{n})$ is 200 bps.

In Case B2, we consider the same range of values for $\alpha$, but now both the division probability and the loss term are length-dependent so that $P_{div}= (a+b\overline{n})^\alpha$ and $y(\overline{n}) = y_0+\overline{n}y_1$. For the purposes of illustration, we fix $y_0$ by taking half the value used in Case B1, and choose $y_1$ so that $2975y_1=y_0$, and when $\overline{n}\approx 3000$, the total $y_0 + \overline{n}y_1$ is the same as the value of $y_0$ in Case B1. If we were to take the more extreme case where $y_0=0$, then the telomere loss term $y_n=ny_1$ would decrease to zero for short telomeres, giving a degenerate approach to senescence.

We partition the ten Cases listed in Table 2 into two groups, according to whether telomere loss depends on telomere length: for Cases A1 and B1 telomere loss is fixed, whereas for Cases A2 and B2 the loss term depends on telomere length. For Cases A1 and A2, we distinguish two types of cells: those which can divide and those which are senescent. However, in Cases B1 and B2 the probability of cell division depends on telomere length and, as in the chromosome model, we distinguish *three* types of cells: those which have just divided $(\phi _{div })$, those which could have divided but did not ($\phi _{pot }$), and those which are senescent ($\phi _{sen}$). To compare Cases B and A, we define the fraction of nonsenescent chromosomes in Case B as $(\phi _{div}+\phi _{pot})=\phi _{nonsen}$.

**Fig. 8:**
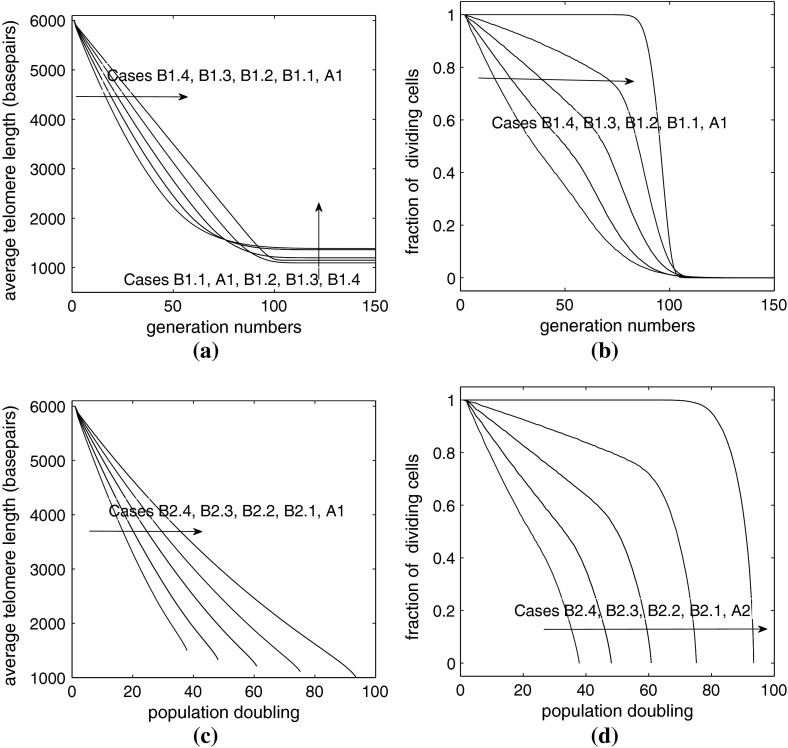
Average of 1,000 simulations, parameters given in Table 2. **a** Average telomere length plotted against generation number. **b** The same data as (**a**), showing the fraction of dividing cells $\phi _{div}(g)$ plotted against generation number. **c** Average telomere length plotted against population doubling. **d** The fraction of dividing cells, $1-\phi _{sen}$, plotted against population doublings

In Fig. 8a we compare Case A1, with various examples from Case B1. Case A1 is the simpler model in which cells always replicate (that is $P_{div}=1$), and telomere is lost at a fixed rate of 200 bps per replication. In Case B1, there is a probability of cell division, $P_{div}=((n-200)/5750)^\alpha$ and the loss rate is constant $y(n)=y_0$ with $240 \le y_0 \le 414$, the precise value depends on $\alpha$ according to Table 2. The plot shows how the average telomere length of the cells changes with generation number for different values of $\alpha$. Before generation 80, Case B1.4 loses telomeres at the fastest rate followed by Cases B1.3, B1.2, B1.1, and A1.

Figure 8a also shows that when the cells reach senescence, Case B1.4 yields cells with the longest telomeres (closely followed by B1.3). Because Case B1.4 has the largest loss term $y(n)=y_0$, it also has the largest critical threshold length. As the generation number increases, Case A1 shows a sharp transition from linear loss (at a relatively slow rate) to senescence. By contrast, Cases B1.1–B1.4 exhibit increasingly smooth and diffuse transitions, from linear loss to a plateau. This is because the rate of loss of telomere declines as the telomeres shorten.

In Fig. 8b we show how the fraction of dividing cells changes with generation number for the different cases. As before, Case A1 shows a sudden transition near generation 100, while the transitions for Cases B1.1–B1.4 are more gradual. In all cases, however, the cells become fully senescent around generation number 100.

The behavior of Cases B2 and A2 is almost identical to that of B1 and A1. However, Fig. 8c, d differ from Fig. 8a, b because we have plotted these results against population doubling ($pd$) instead of generation number. If we denote the total number of cells in the population by $N(g)$ we have, from (3)

5 $$\begin{aligned} N(g+1) = (1+\phi _{div}(g)) N(g) , \quad \text{ and } \quad pd = \log _2 \left( \frac{N(g)}{N(0)} \right) . \end{aligned}$$

Thus, even though $g$ can increase without limit, $pd$ will reach a maximum when $\phi _{div}=0$, that is, when all cells are senescent. Comparing the results in Fig. 8c, d we observe that Case B2.4 reaches senescence with the smallest number of population doublings, that is the smallest total number of cells, and Case A2 yields the largest final population size.

## Modelling Werner’s Syndrome

In this section, we generalise the above models to describe Werner’s syndrome. We start with models in which each cell contains a single chromosome and where Werner’s syndrome manifests itself *via* the deletion of extra basepairs from each telomere. We then upscale these models to study cells that contain many chromosomes.

### Development of the Chromosome Model

As above, we denote by $m$ and $n$ the lengths of the telomeres at each end of the chromosome. During replication, $y$ basepairs are lost from one of the daughter chromosomes due to normal aging. We introduce an additional degree of stochasticity into the replication process by defining a probability $p_w$ that during replication, one of the daughter chromosomes suffers a *further* telomeric loss of $x$ bps due to Werner’s syndrome.

To allow a fair comparison of different values of $p_w$ we choose $x$ and $p_w$ so that the expected loss due to Werner’s syndrome is the same in all cases, that is $xp_w=$ constant (see Table 3 for a list of the values considered). For comparison, we also present results for the case $p_w=x=0$ which corresponds to normal aging.

**Table 3:** Values of $p_w$ and $x$ used in simulations of Werner’s syndrome

$p_w$	0	0.2	0.4	0.6	0.8	1
$x$	0	1000	500	333	250	200

Note $xp_w=200$ in all cases except the first

**Fig. 9:**
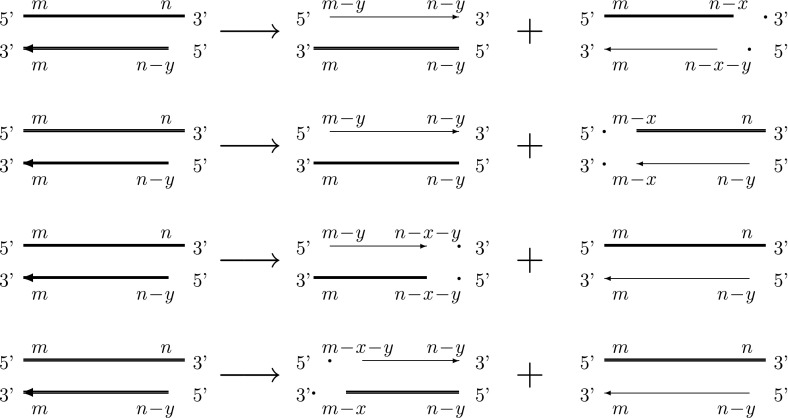
The four ways in which telomere shortening can occur with Werner’s syndrome and normal aging. The *thick lines* represent the template (parent) strands and the *thin lines* represent the replicated strands of the template in the daughter chromosomes. The *arrows* show the directions of replication

In more detail, at the start of replication, for each cell, (and on each generation), we generate a random number $r$ from a uniform distribution over [0,1). If $r<p_w$ then replication occurs as described below; otherwise, if $r \ge p_w$, then normal replication occurs as in (1). The four ways in which an additional deletion can occur are depicted in Fig. 9. If we assume that the probability $p_x$ of each of these replication rules are the same, then Fig. 9 can be written as

6a $$\begin{aligned} \left( \begin{array}{cc} m &{} n\\ m &{} n-y \end{array}\right)&\rightarrow \left( \begin{array}{cc} m-y &{} n-y\\ m &{} n-y \end{array}\right) + \left( \begin{array}{cc} m &{} n-x\\ m &{} n-x-y \end{array}\right) , \nonumber \\&\qquad \mathrm with probability \quad p_x={\frac{1}{4}},\end{aligned}$$

6b $$\begin{aligned} \left( \begin{array}{cc} m &{} n\\ m &{} n-y \end{array}\right)&\rightarrow \left( \begin{array}{cc} m-y &{} n-y\\ m &{} n-y \end{array}\right) + \left( \begin{array}{cc} m-x &{} n\\ m-x &{} n-y \end{array}\right) , \nonumber \\&\qquad \mathrm with probability \quad p_x={\frac{1}{4}},\end{aligned}$$

6c $$\begin{aligned} \left( \begin{array}{cc} m &{} n\\ m &{} n-y\end{array}\right)&\rightarrow \left( \begin{array}{cc} m-y &{} n-x-y\\ m &{} n-x-y \end{array}\right) + \left( \begin{array}{cc} m &{} n\\ m &{} n-y\end{array}\right) , \nonumber \\&\qquad \mathrm with probability \quad p_x={\frac{1}{4}},\end{aligned}$$

6d $$\begin{aligned} \left( \begin{array}{cc} m &{} n\\ m &{} n-y\end{array}\right)&\rightarrow \left( \begin{array}{cc} m-x-y &{} n-y\\ m-x &{} n-y \end{array}\right) + \left( \begin{array}{cc} m &{} n\\ m &{} n-y\end{array}\right) ,\nonumber \\&\qquad \mathrm with probability \quad p_x={\frac{1}{4}}. \end{aligned}$$

In normal aging, chromosomes stop replicating when the telomere length reaches a critical value, which we have assumed to be zero. However, in Werner’s syndrome, senescence is less well defined, since it depends on whether the extra deletion occurs in the same place as the loss due to normal aging, or at a different end. For example, consider the case $n=y$, for which the telomere can be schematised as $$\left( \begin{array}{cc} m &{} y \\ m &{} 0 \end{array} \right) $$. Strictly speaking, (6) suggests that there are four possible outcomes. However, rules (6a) and (6c) lead to physically unrealistic telomere lengths; we will assume that these outcomes cannot occur, and in such cases the parent chromosome remains undivided. Thus, when one end of a Werner’s syndrome chromosome nears the critical telomere length, the three possible outcomes following replication are

7a $$\begin{aligned} \left( \begin{array}{cc} m &{} y\\ m &{} 0\end{array}\right)&\rightarrow \left( \begin{array}{cc} m-y &{} 0\\ m &{} 0 \end{array}\right) + \left( \begin{array}{cc} m-x &{} y\\ m-x &{} 0 \end{array}\right) ,\nonumber \\&\qquad \mathrm with probability \quad p_x = {\frac{1}{4}},\end{aligned}$$

7b $$\begin{aligned} \left( \begin{array}{cc} m &{} y\\ m &{} 0 \end{array}\right)&\rightarrow \left( \begin{array}{cc} m-x-y &{} 0\\ m-x &{} 0 \end{array}\right) + \left( \begin{array}{cc} m &{} y\\ m &{} 0 \end{array}\right) ,\nonumber \\&\qquad \mathrm with probability \quad p_x = {\frac{1}{4}} ,\end{aligned}$$

7c $$\begin{aligned} \left( \begin{array}{cc} m &{} y\\ m &{} 0\end{array}\right)&\rightarrow \left( \begin{array}{cc} m &{} y\\ m &{} 0\end{array}\right) , \quad \mathrm with probability \quad p_x = {\frac{1}{2}}. \end{aligned}$$

Equation (7a) corresponds to (6b) in the case $n=y$, and similarly, (7b) corresponds to (6d).

To summarize, when a Werner’s syndrome deletion occurs, replication follows one of Eqs. (6a–6d), each outcome occurring with probability $p_x=1/4$, provided that the telomere length exceeds the critical telomere length. If, during replication, telomere loss would result in a length below the threshold, then replication will not occur, as in (7c).

We use the same initial data as in earlier sections, that is, we start simulations with a single chromosome three of whose telomeres have lengths $m = n = 6,000$ bps, and one of length 5,800 bps. We assume a loss of telomere of $y=200$ bps per replication due to normal aging, as in Case A1, and we continue to define as senescent those chromosomes whose telomeres are less than 200 bps in length. We use the passaging method described in Sect. 2.1.

### Results for the Chromosome Model of Werner’s Syndrome

Our aim is not only to contrast normal aging with Werner’s syndrome, but also to compare alternative characterisations of Werner’s syndrome; that is, we compare frequent losses of a small amount of telomere (large $p_w$, small $x$) with rare losses of larger amounts (small $p_w$, larger $x$).

**Fig. 10:**
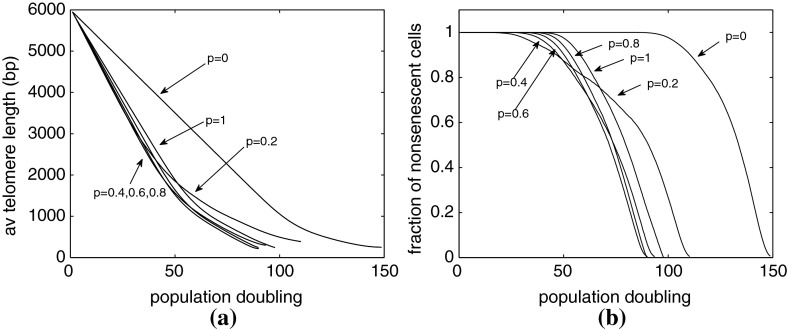
**a** Average telomere length plotted against population doubling for simulations of Werner’s syndrome. **b** The proportion of nonsenescent cells plotted against population doubling for Werner’s syndrome simulations. For each value of $p=p_w$, we present results obtained by averaging over 2,000 simulations. For parameter values, see Table 3

Figure 10a shows how the average telomere length varies with population doubling and the probability of undergoing a Werner’s deletion increases. We observe that when $p_w>0$, all cases yield similar results, because the average rate of telomere loss per replication event is the same (200 basepairs). For Werner’s syndrome, that is, $p_w>0$, as the number of population doublings increases, the average telomere length decreases linearly for the first 50 pds (in contrast to 100 pd when $p_w=0$). There ensues a second period, of about 50 pds, during which the rate of telomere loss occurs at a slower rate, until, by approximately pd 100, the entire population becomes senescent.

**Fig. 11:**
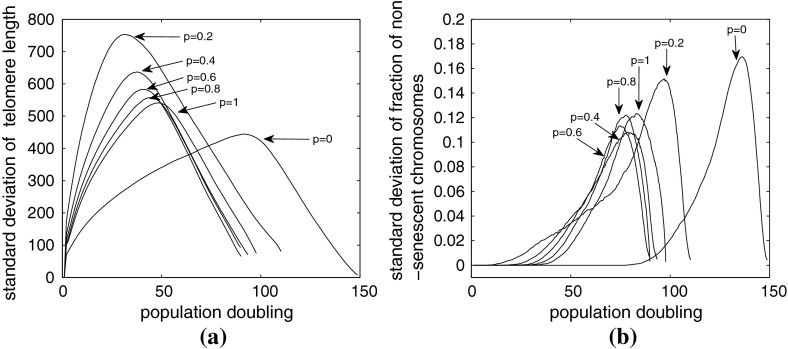
**a** The standard deviation of the telomere length plotted against population doubling, the same data as in Fig. 10a. **b** The standard deviation of the fraction of nonsenescent chromosomes plotted against population doubling, again, the same data as in Fig. 10b

We summarize the data from Fig. 10 in Table 4. As might be expected, the smallest pd at which senescence occurs is for the system with the largest deletion ($x=1000$), whereas the latest occurs for the system with the smallest deletion ($x=200$) at pd 35. Note that this is not where the change in gradient in the curves in Fig. 10a occurs. As expected, Table 4 also reveals that Werner’s syndrome cells reach senescence faster than normal aging ($p_w=0$); all Werner’s syndrome populations become senescent after 93$\pm$4 population doublings apart from the case $p_w=0.2$, which takes only slightly longer. The final dataset shown in Table 4 is the average pd at which the whole system becomes senescent. This shows that the cases with rare but massive telomere deletions due to Werner’s syndrome become senescent with longer telomeres than those cases with more frequent but shorter deletions. Also as noted above, cases with large but rare deletions actually yield more pds than the smaller frequent deletions. Figure 10b shows how the fraction of nonsenescent chromosomes $\phi _{div}$ varies with pd and with $p_w$. The graphs are similar for $p_w>0$, with perhaps the case of $p_w=0.2$ exhibiting more interesting behavior. All curves depart from $\phi _{div}=1$ at the population doubling (pd) where senescence starts (central column of Table 4); however, we observe senescence of the *whole* population at approximately the same pd for all $p_w>0$. When $p_w = 0.2$ the proportion of dividing cells initially declines only slowly; while later, around pd 85, there is a transition to a more rapid increase in senescence. The case with $p_w=0.2$ reaches senescence a few pds later than those cases with higher values of $p_w$, as can also be seen in Fig. 11.

**Table 4:** Summary of the key data from Figure 10

$p_w$	$x$	pd at which senescent cells first appear	Final number of population doublings	Mean telomere length at senescence
0	0	85	150	250
0.2	1000	11	110	387
0.4	500	20	93	300
0.6	333	27	89	216
0.8	250	31	89	247
1	200	35	97	244

In Fig. 10, we present the averaged results, with no measure of the degree of variability within the simulation data. In Fig. 11a we show how the standard deviation of the average telomere length varies with pd. For Werner’s syndrome ($p_w>0$), the larger $x$ is (smaller $p_w$), the larger the standard deviation of average telomere length throughout the simulation. The standard deviation starts at zero as all simulations have the same initial data of a single of chromosome. Initially, the standard deviation increases with the square root of pd as one would expect, reaching a peak, which occurs earlier for larger deletions, $x$. Comparing these figures with Table 4 we note that the peak occurs just after the chromosomes start to senescence. The standard deviation then decreases linearly with pd, approaching zero when the entire population reaches senescence.

Figure 11b shows how the standard deviation of the fraction of dividing (nonsenescent) chromosomes varies with pd. At early times the standard deviation is identically zero since all chromosomes are dividing in all simulations. Once some senescent chromosomes appear in some simulations (see Table 4), the standard deviation increases gradually, reaching a maximum shortly before total senescence of the whole population occurs, and then the sd decreases rapidly as the entire population becomes senescent. The shape of curves is broadly similar for $0.4\le p_w\le 1$, while $p_w=0.2$ is a little more like that of normal aging, having a gradual increase at intermediate times with a significant acceleration just before the maximum, which occurs at larger pd than other cases of Werner’s syndrome ($p_w>0.2$).

### Results for the Stochastic Cell Model of Werner’s Syndrome

We now use the techniques outlined in Sect. 3.1 to extend the single chromosome model to a cell-level model, with $N=46$ chromosomes per cell. We apply the replication rules (6–7) with probability $p_w$ to each chromosome in each cell, and use Eq. (1) otherwise. If one chromosome is unable to divide, due either to normal aging or Werner’s syndrome, the cell is unable to divide, but is retained in the population and may attempt to divide in a subsequent generation.

We record the average telomere length and the fraction of dividing cells over each generation. We find that both quantities evolve in a similar fashion to the single chromosome model; the main difference is that a cell with $N=46$ chromosomes becomes senescent earlier, and with much longer telomeres, than the single chromosome model.

**Fig. 12:**
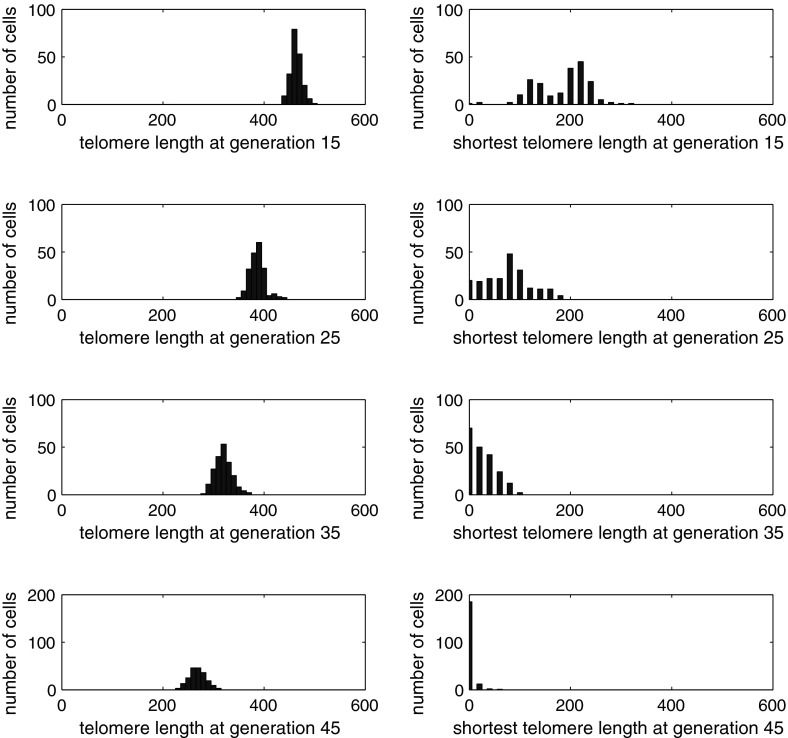
Histogram of average telomere length (*left*) and shortest telomere length (*right*) of the cell in a simulation of Werner’s syndrome with $p_w=0.2$ and $x=1,000$. The horizontal scale in graph is reduced by a factor of 10, that is, $0 \le n \le 6,000$ bps

In order to illustrate the distribution of telomere lengths in Werner’s syndrome, we record both the *average* telomere length and the *shortest* telomere of 200 cells in one simulation, every five generations from 15 to 45. The resulting data, presented in Fig. 12, shows that, as the generation number increases, the mean telomere length steadily reduces while the distribution of telomere lengths slowly spreads out. The distribution of the *shortest* telomere lengths exhibits distinct behavior: at generation 15 the distribution is bimodal, but as the generation number increases, the distribution becomes unimodal again (the initial conditions having been unimodal) and approaches zero telomere length. The bimodality is due to a significant number of cells having suffered a massive Werner’s deletion, while others have undergone only normal aging; for example, at later times, some telomeres may have undergone three or four massive deletions and others only one or two. At generation 45, the shortest telomere length of almost all cells is close to zero and so they are senescent. The telomere lengths of many other chromosomes in the cell are still sizable, so the *average* telomere length remains fairly large, specifically about 2500 bps. Thus, if data were available on the distribution of telomere lengths in replicating cells and in the population of senescent cells, it would be possible to determine roughly the size of deletions ($x$) and the probability of additional Werner’s deletions occurring ($p_w$).

## Discussion

In this paper, we have developed chromosome- and cell-level models of telomere loss during replication and compared alternative models of telomere shortening during replication. In this section, we compare our results with experimental data and simulations obtained by other groups.

**Fig. 13:**
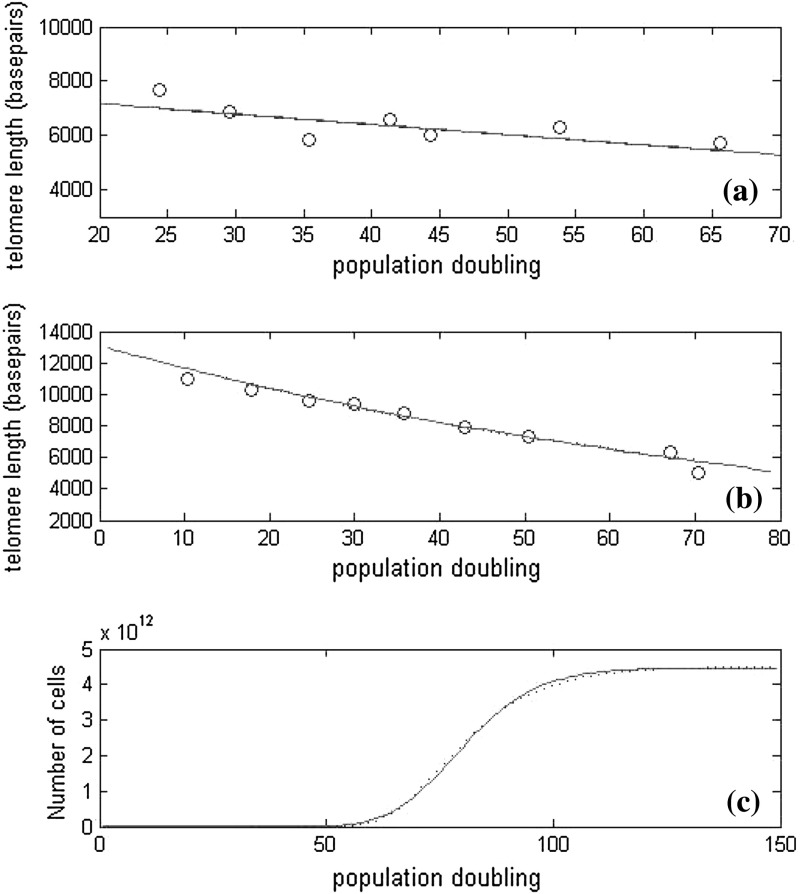
**a** Average telomere length plotted against population doubling; *solid line* shows our simulation results, the *dotted line*, the fit of Levy et al. (1992), *circles* show the experimental data from Levy et al. (1992). **b** Again, average telomere length is plotted against population doubling; the *circles* show experimental data from Zhang et al. (2000), the *dotted line* shows the results of Buijs et al. (2004) and the *solid line* is our simulation data (Case A1). Note that the *solid* and *dotted lines* coincide. **c** The number of cells plotted against generation number, the *solid line* indicates results from our Case B1 and the *dotted line* is a fit to a Gompertzian growth model. All three plots demonstrate the close agreement between our simulation results, those of previous models, and experimental data

The first case we consider is that in which a fixed amount of telomere is lost during chromosome/cell replication, and in each timestep the cells divide if their telomeres are long enough to allow replication (Case A1). Levy et al. (1992) modeled telomere shortening of chromosomes with a constant telomere loss caused by the “end-replication” problem. Their model predicted average telomere length decreasing linearly with generation number. In our first model, we see that the average telomere length of the chromosomes in the cell decreases linearly as the population doubling or generation numbers increase. This behavior only occurs before appreciable numbers of cells become senescent, and this result is consistent with the work of Levy et al. (1992). In Fig. 13a, we compare our results with the data used by Levy et al., and find that our simulation results are identical to Levy’s work (the two curves are indistinguishable). This is as expected since their model is fundamentally the same as our Case A1. Our Case A2 corresponds to the situation in which the amount of telomere loss during chromosome replication is dependent on the length of the telomere. Buijs et al. (2004) showed that the shortening process was dependent on telomere length. They fitted experimental data of the distributions of telomere lengths at various stages of the shortening process to normal, log normal and Weibull distributions, with a loss term that is linearly dependent on telomere length as in (2). Figure 13 b shows that our simulation results are consistent with the results of Buijs et al. and with the experimental data which they used.

In Case B1, cell division is assumed to be a random process with the probability of a cell or chromosome replicating being dependent on telomere length. The amount of telomere lost per replication is fixed. Portugal et al. (2008) developed a similar stochastic model, though their focus was on the growth rate of the cells, rather than average telomere length. They predicted Gompertzian growth in the cell population. Figure 13c shows that the total cell population in our simulations can also be fitted by a Gompertzian growth law. This has the form $N=a \exp ( -b \mathrm{e}^{-ct})$ where $N$ is the total number of cells, with $a,b,c>0$; such a growth law satisfies the differential equation $\mathrm{d}N/\mathrm{d}t = c N \log (a/N)$. While $N$ grows exponentially at early times, the term $\log (a/N)$ decreases linearly, and then saturates at zero as $N$ increases to $a$. In our model we have $\mathrm{d}N / \mathrm{d}t= \gamma N \phi _{div}(t)$, with $\phi _{div}$ exhibiting similar behavior, namely linear decay to zero, although this follows a period during which $\phi _{div}=1$. Thus, Gompertzian growth is broadly consistent with our model.

While models A1, A2, and B1 have been used by other researchers, Case B2 is new. In this case, we combined telomere length-dependent loss with a model in which the probability of replication is also dependent on telomere length. When the parameters are chosen appropriately, all earlier models can be recovered as special cases. In Eq. (2) which governs how the number of basepairs lost and the probability of division depends on telomere length, $n$; we fix $y_1=0$ and $\alpha =0$ for Case A1, so that the amount lost is length-independent ($y(n)=y_0$) and all nonsenescent cells divide, $P_{div}=1$. For A2, we fix $\alpha =0$ so that $P_{div}=1$ but the loss term $y(n)=y_0+y_1n$ is left general; and for B1, we take $y_1=0$ so that the loss term is constant, $y(n)=y_0$, but the probability of division is left general.

While Levy et al. (1992), Portugal et al. (2008) and Buijs et al. (2004) have considered populations of independent chromosomes undergoing replication following Cases A1, A2, and B1, we have generalized their results to investigate replication of *cells* which contain $N=46$ chromosomes. We consider how the fraction of dividing cells (chromosomes) changes as the generation number increases. We notice that chromosomes become senescent when their telomeres have reduced to about 150–250 bps in the model where chromosomes replicate independently. However, in the model with 46 chromosomes per cell, when the cells reach senescence, the telomeres have lengths of about 1,150–1,500 bps per chromosome. This is because if the length of even *one* chromosome falls below the critical value then all other chromosomes, which contain longer telomeres, also cease dividing. Thus, if we consider an increased number of chromosomes per cell, the average telomere length at which they become senescent will also increase.

**Fig. 14:**
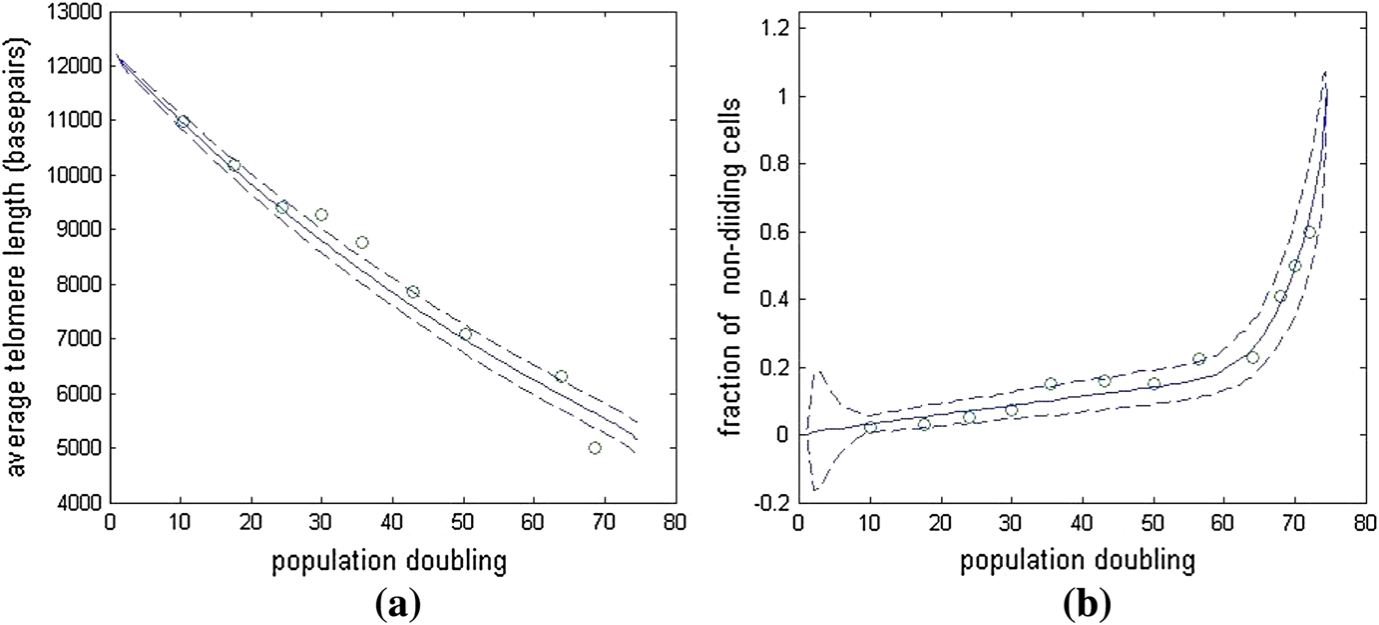
Average results of 200 simulations of the cell-level model, Case B2, in which both the number of base pairs lost in replication, and the probability of a cell dividing are dependent on telomere length, via (2), with values given by $Y(n)=10+0.043n$ and $P_{div}(n)=(n/12200 -0.03)^{0.25}$ respectively. **a** Mean telomere length plotted against population doubling, (*solid line*), the *dashed lines* indicate two standard deviations above and below the mean. *Circles* show the experimental results of  Zhang et al. (2000). **b** The mean fraction of nondividing cells plotted against population doubling, (*solid line*), the *dashed lines* indicate two standard deviations above and below the mean. The anomalously large standard deviation in the first few population doublings is due to there being few cells present in the simulations at early times. Since the simulations start with one cell and in the later stages retain only 200 cells, it takes eight generations for the population to reach 200 cells. Note in particular, the good agreement in **b** where there is a sudden sharp increase in the fraction of nondividing cells around pd 65. This is due to the majority of cells in the population becoming senescent around this population doubling

Figure 14 demonstrates that our stochastic simulation results for the cell-level model of Case B2 can be fitted to the experimental data of  Zhang et al. (2000). Both the average telomere length and the fraction of replicating (nondividing) cells are plotted against pd. We use our model to estimate the rate of telomere loss and the probability of a cell division. We use an initial telomere length of 12,200 basepairs, the amount of telomere loss is $Y(n)=10+0.043n$ and the probability of a cell dividing is $P_{div}(n)=(n/12200 -0.03)^{0.25}$.

## Conclusions

In this paper, we have developed a series of increasingly complex mathematical models to study telomere shortening in a population of healthy aging chromosomes, and in a population of cells each of which contains $N=46$ chromosomes. We have also generalized these models to investigate the effects of Werner’s syndrome. In normal aging, $y$ basepairs are lost from a telomere in each replication; in Werner’s syndrome, there is an additional loss of $x$ basepairs at one or other end of one of the daughter chromosome in some or all replication events. In each model, we have simulated systems in which the amount of telomere lost is fixed, or is length-dependent; cases where cells/chromosomes divide whenever possible, or when there is a probability of division which depends on telomere length. For Werner’s syndrome, we have considered cases where a small amount of telomere is lost every replication, and cases where there is a probability of a much larger deletion. We expect the latter case to be the more relevant, since it leads to the more gradual reduction in the proportion of proliferating cells, note that the $p=0.2$ curve in Fig. 10 provides a better to fit to Fig. 2 of Faragher (2004) than any of the $p$ values.

Comparing the results of Werner’s syndrome and normal aging, we have seen that Werner’s syndrome cells (chromosomes) reach senescence significantly earlier than normal cells, which confirms that Werner’s syndrome accelerates the aging process matching a characteristic clinical feature of Werner’s syndrome, namely the premature appearance of aging (Yamamoto et al. 2003). Another significant observation from our results is that when cells with Werner’s syndrome become senescent, they contain *longer* telomeres than cells subject to normal aging alone, and a broader range of telomere lengths. Figure 12 indicates that the shortest telomere length in the cell reaches the critical value while the average telomere length is still quite long. These results are consistent with the explanation of Chang (2005), who predicted that populations of cells with Werner’s syndrome will contain some very short telomeres with the majority retaining longer telomeres. Thus, in Werner’s syndrome, we observe not only an accelerated telomere shortening, but also a higher variability in telomere lengths causing premature senescence. Both these properties contribute to accelerated aging that characterizes Werner’s syndrome.

This greater variance is maximised with the largest values of loss and the smallest probability of extra Werner’s deletions. This case also gives rise to a multi-peaked distribution of the *shortest* telomere lengths in each cell.

In the case of normal aging, with fixed loss and all cells dividing whenever their telomeres are sufficiently long we see a linear decrease in average telomere length, with a sharp transition to senescence. In the more general cases where telomere loss and/or the probability of division are length-dependent, the population’s progression into senescence and transition from telomere shortening to a plateau are much smoother. We note that the total number of cells/chromosomes in the population fits well to a Gompertzian growth curve, as shown in Fig. 13c.

Finally, in Sect. 5 we have compared our models against experimental data from the literature, and the models of other theoreticians. We have plotted the average telomere length against population doubling. We have found good fits both to the models of  Levy et al. (1992) (see Fig. 13) and  Buijs et al. (2004), and the data of  Zhang et al. (2000). The fit shown in Fig. 14 yields only a weakly length-dependent probability of replication, $P_{div} = (n/12200-0.03)^{0.25}$ which has the range $0.785 < P < 0.992$ for $5000<n<12200$, but a strongly length-dependent telomere loss term of the form $Y(n) = 10+0.043n$ giving the range $225 < Y < 535$.

In another paper Qi et al. (2013), we use asymptotic techniques to investigate how, for normal aging, quantities such as the telomere length, and the fraction of senescent cells vary with time (population doubling or generation number), as well as the shape of the distribution of telomere lengths. This theory describes the kinetics of the rules simulated here by a discrete dynamical system which, using techniques from asymptotic analysis, can be approximated by a partial differential equation, for which the exact solutions are available in a number of cases. This work builds on the papers of  Antal et al. (2007) and   Hirt et al. (2013). Further simulation results and theoretical analysis is available in Qi (2011). In future work Qi et al. (2014), we propose to model and analyze the effect of the telomere-lengthening enzyme telomerase, using both simulations and asymptotic approximations.

## Abbreviations

bps: Basepairs
pd: Population doubling
sd: Standard deviation
WRN: Gene responsible for Werner’s syndrome
